# Effects of probiotic supplementation on testosterone levels in healthy ageing men: A 12-week double-blind, placebo-controlled randomized clinical trial

**DOI:** 10.1016/j.conctc.2024.101300

**Published:** 2024-04-25

**Authors:** Lennart Ljunggren, Eile Butler, Jakob Axelsson, Mikael Åström, Lars Ohlsson

**Affiliations:** a Malmö University, Faculty of Health and Society, Department of Biomedical Science, SE-20506, Malmö, Sweden; b Atlantia Clinical Trials, Heron House, Blackpool Retail Park, Cork, T23R50R, Ireland; c BioGaia AB, Mobilvägen 10, SE-223 62, Lund, Sweden; d StatCons, Högerudsgatan 8 B, SE-21618, Limhamn, Sweden

**Keywords:** Probiotics, Testosterone, Ageing men, *Limosilactobacillus reuteri*, Lipids, Triglycerides

## Abstract

Levels of the male sex hormone testosterone are generally stable in the age interval 20–70 years, but several studies indicate an earlier, age-dependent decline. Testosterone deficiency is often underdiagnosed and under-treated, but replacement therapy has nonetheless increased during the last couple of years. Owing to possible negative side effects, alternative treatments have been investigated, including different supplementation protocols. The aim of this study was to investigate the effect of probiotic supplementation on the testosterone level in healthy men aged between 55 and 65. Hence, 12 weeks randomized, double-blinded, placebo-controlled trial was conducted to investigate the effect on testosterone levels following supplementation of the recognized probiotic *Limosilactobacillus reuteri* ATCC PTA 6475 on testosterone levels, using high-, low- or placebo treatment. Venous blood samples were collected at baseline, 6 and 12 weeks, for analysis of bloodwork, lipid profile, hormones, and electrolytes. Subjects were also asked to complete a questionnaire. The supplementation had no effect on testosterone levels, neither using high- or low dose, nor placebo. However, a significant decrease of triglyceride levels was observed in the high-dose group. No other parameters showed any significant change. The present study does not support the hypothesis that a probiotic supplementation can increase testosterone levels in ageing men.

## 1 Background

Testosterone is the primary male sex hormone and is pivotal in determining male sexual differentiation, development, and maturation through the entire lifespan of the individual. Testosterone plays a key role in the development of male reproductive tissues such as testes and prostate and promotes secondary sexual characteristics such as increased muscle and bone mass, and the growth of body hair. Testosterone is synthesized by Leydig cells in the testes in the presence of luteinizing hormone (LH). Circulating testosterone is mostly attached to the proteins sexual hormone binding globulin (SHBG) and albumin, while some circulates as free testosterone.

In males, serum testosterone levels peak around the age of 20 and usually remain stable until over the age of 70 [1]. However, there is a large amount of literature arguing that age is a factor in testosterone decline. Thus, several cross sectional and longitudinal studies have indicated age associated declining serum testosterone levels associated with age [[2], [3], [4]].

Travison et al. observed that there was a substantial decline in total- and free testosterone associated with ageing alone, however other factors were also associated with an accelerated decline including an increase in Body Mass Index (BMI) and the loss of a spouse [3]. It has been shown that the total testosterone level in obese men is lower than in men of normal weight of the same age, partly due to a lower SHBG levels in obesity.

Even though other factors, such as smoking, depression, or marital status have also been linked to a decrease in testosterone levels, an age-related impact on testosterone levels remains even after adjusting for these factors [2].

Low levels of serum testosterone are associated with specific symptoms. The most easily recognizable symptoms of this in older men are usually a decrease in muscle mass, strength and bone mass and an increase in central body fat. Other symptoms like decrease in libido and sexual desire, loss of memory, insomnia and a decreased sense of well-being are difficult to measure but are likely attributable to low testosterone [5]. Testosterone deficiency is frequently overlooked and inadequately treated since the symptoms reported by men with low androgen levels are often dismissed and attributed to the natural aging process. Bhasin et al. suggested that low testosterone affects an estimated 2–4 million men in the US alone and in testosterone supplementation has increased over the past several years [6].

Testosterone replacement therapy is the standard therapy for young adults, aiming to restore normal range hormone levels and alleviate the symptoms associated with deficiency. However, there are challenges and side-effects, associated with replacement therapy [5].

Due to the controversy regarding testosterone replacement therapy and potential side effects, other alternatives are being investigated.

Some research has investigated the links between probiotic supplementation and their effects on various hormones, such as corticosterone, oxytocin, and testosterone. One study with probiotics showed that *L. rhamnosu*s (JB-1) had an effect in reducing stress induced corticosterone in a mouse model of anxiety [7]. However, in a human study, it was shown that supplementation with probiotics was not superior to placebo in the reduction of symptoms of anxiety or inflammatory markers, showing the difficulties in translating animal models into studies on humans [8].

*Limosilactobacillus reuteri* (*L. reuteri*) is one of the most widely investigated probiotic bacterium commercially available today and beneficial clinical effects have been shown in gastro-intestinal health, immunity and even the gut-brain axis [[9], [10], [11]].

Regarding L. *reuteri* and the gut brain axis, it was shown that after supplementation with *L. reuteri* ATCC PTA 6475 in mice, there was a significant acceleration in wound healing and hair re-growth could be linked to the upregulation of oxytocin [12]. The same bacterial strain has previously been tested in a higher dose in a human clinical study investigating the effect on bone mass density in post-menopausal women, showing that the supplementation was beneficial in reducing bone loss [13].

In 2014, Poutahidis et al. showed, using mouse models, that *L. reuteri* ATCC PTA 6475 consumption increased testicular weight as well as serum testosterone and counteracted age-related testicular atrophy [14].

Probiotic organisms may affect different hormones and offer practical options for management of disorders frequently associated with normal aging [15,16].

The aim of this study was to evaluate the potential effect of probiotic dietary supplementation on testosterone levels (and other relevant testosterone related hormones) in healthy middle-aged and elderly men and the effect on other biomarkers associated with testosterone.

## 2 Materials and methods

### 2.1 Study products (IP)

The two active study products contained *L. reuteri* ATCC PTA 6475, Vitamin D_3_ and maltodextrin in a capsule. Active study product *Low dose* contained a dose per capsule of *L. reuteri* ATCC PTA ≥5 × 10^8^ colony-forming units (CFU) and vitamin D_3_ 5 μg. Study product *High dose* contains a dose per capsule of *L. reuteri* ATCC PTA ≥5 × 10^9^ CFU and vitamin D_3_ 5 μg. *L. reuteri* ATCC PTA 6475 is a naturally occurring strain of human origin [17]. The placebo product (CP) was of identical formulation, Vitamin D_3_ (5 μg/capsule) and maltodextrin without *L. reuteri* ATCC PTA.

### 2.2 Study design and dose groups

The study was a double-blind, randomized, three-arm placebo-controlled parallel-group study with the primary objective to evaluate if daily supplementation with *L. reuteri* ATCC PTA 6475 can influence testosterone levels over 12 weeks of intervention.

The study consisted of a combined screening and randomization and baseline sample collection visit, and two follow up assessments for 90 days (follow-up 44- and 90-days post start of treatment).

Healthy male subjects, 50–65 years of age, were recruited using advertising (e.g., in social media). All subjects were willing and able to give informed consent for participation in the study, capable of understanding and complying with the requirements of the study, had no known underlying medical conditions and no history of androgen or testosterone supplementation 6 months prior to study start. Subjects who presented with antibiotics or probiotic supplements use 4 weeks prior to study start, subjects who presently have or have had cardiovascular issues in the past 2 years, subjects who have a Body Mass Index of <18 or >30, had known elevated Prostate Specific Antigen level, a history of prostate or testicular cancer, had nicotine or alcohol abuse were excluded from the study. Brief oral information about the study was given and pre-defined pre-screening questions asked.

During the screening and randomization assessment (visit 1), full information about the study was given and informed consent obtained. Information regarding demographics, age, weight, height, occupation/retired, marital status, children, sleep, alcohol intake and exercise levels was collected, and the subjects filled out the Ageing Males Symptoms (AMS) Questionnaire [18,19]. Subjects were screened for eligibility as per the pre-defined eligibility criteria, as specified in the ethical application for the study.

Eligible and consenting subjects were randomized to intervention with either Low dose, High dose or placebo. The subjects were provided with study product or placebo taken twice daily (for a total daily dose of 1 × 10^9^ CFU (Low dose) and 1 × 10^10^ CFU (high dose) for eight weeks and a diary for checking compliance and capturing adverse events (AEs).

The subjects were contacted 1–3 days after randomization to confirm that the first IP or CP had been taken. At visits 2 and 3 (44- and 90 days post randomization, respectively), the subjects were asked to answer the AMS questionnaire. Information on AEs, parallel medications, use of restricted products, and compliance with instructions for IP intake was collected.

### 2.3 Blood samples

Blood samples were taken at three separate timepoints: at baseline, 44- and 90 days (end of study). The following markers were analyzed in peripheral blood and plasma at the Clinical chemistry department, SUS, Malmö, Sweden:

Testosterone, SHBG, Dehydroepiandrosterone sulphate (DHEAS), Thyroid Stimulating hormone (TSH), Thyroxine (T4), Bilirubin, Insulin, Luteinizing hormone (LH), Prostate-Specific Antigen (PSA), Follicle Stimulating Hormone (FSH), C- reactive protein (CRP), HBA1c, Albumin, Cholesterol, High Density Lipoprotein (HDL), Low Density Lipoprotein (LDL), Triglycerides, Hemoglobin, Calcium, Potassium, Sodium, Creatinine and Cystatin-c/pt-EGFR.

### 2.4 Statistics

The statistical analysis of the efficacy objectives follows the intention to treat (ITT) principle, implying that all randomized subjects who have taken at least one dose of the study product should be included in the ITT analysis.

The Wilcoxon signed rank test was used for comparing a change over time within each group for continuous and ordered categorical data and the Binomial test used for binary data.

The Wilcoxon rank sum test was used for comparing the groups for continuous and ordered categorical data and the Fisher's exact test was used for binary data. Data from all variables are presented by means of descriptive statistics. All presented p-values and confidence intervals are two-sided and nominal.

### 2.5 Ethical aspects, recruitment, and randomization

All subjects provided written informed consent prior to participating in this investigation. This study was conducted according to the guidelines of the declaration of Helsinki and approved by the Ethical Approval Agency in Sweden, given the number DN 2020-01611. The trial was registered at www.clinicaltrials.gov (NCT-04577625). Participants for the study were recruited via CTC Clinical Trial Consultants AB, Uppsala, Sweden. More than 60 men volunteered for eligible testing and 57 were included in the study program. Subjects were randomized into three sequentially numbered equal groups. The randomization record was held by Skåne university clinic (SUS) and handed over for statistical analysis after complete data collection.

## 3 Results

A total of 57 subjects were initially enrolled in the study but only 49 subjects completed according to the ITT principle. The criteria for ITT were (1) correctly included (2) randomized (3) at least on efficacy observation post randomization (4) have taken at least one dose of the study medication. At the clean file meeting it was decided that four of the initial subjects should be excluded from the efficacy analysis using the ITT analysis set, since they did not meet the study criteria. The remaining four subjects were excluded as they did not meet the ITT criteria. They did not take the supplement as instructed or they did not fulfill the blood sampling. One further subject was excluded from the ITT, due to suspected hypogonadism, manifested as extremely low testosterone and high FSH and LH levels.

There was no significant difference between the different groups concerning age or BMI (see Table 1).

**Table 1:** Baseline characteristics of participants.

	Placebo (n = 18)	Low dose (n = 14)	High dose (n = 15)
Age (year)	57.5 ± 4.3	57.3 ± 3.9	56.9 ± 3.3
BMI (kg/m^2^)	25.9 ± 2.4	26.7 ± 2.1	26.4 ± 2.0

**Fig. 1:**
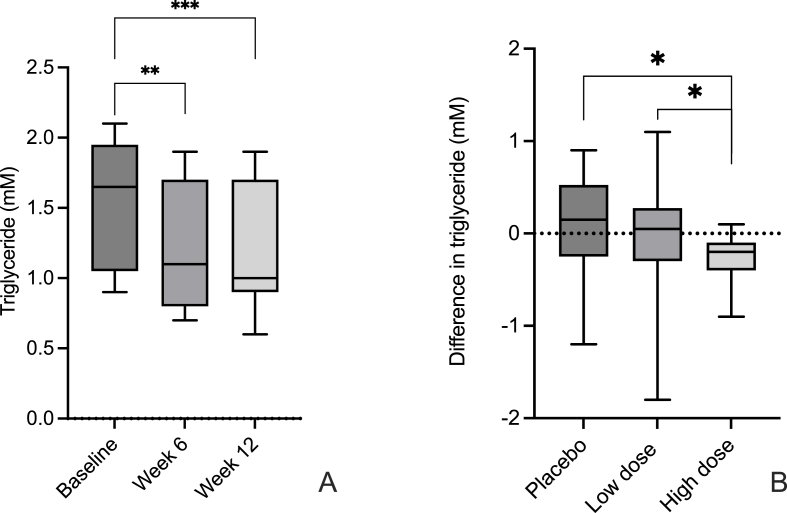
Change in triglyceride concentrations in the high dose group at different time points (A) and between different groups from base line to end point (B). Wilcoxon sign rank test. * = P ≤ 0.01, ** = P ≤ 0.005, *** = P ≤ 0.0001.

Table 2 shows the biochemical characteristics of the three different groups. No significant between-group differences were observed at baseline regarding the investigated parameters. The triglyceride concentration was significantly decreased in the high dose group, by more than 20 % during the first 6 weeks, from 1.54 to 1.22 mM (p = 0.003) and the triglyceride concentration was further decreased after 12 weeks to 1.20 mM (p = 0.0004) as illustrated in Fig. 1A. After 12 weeks, the triglyceride concentrations are different also between the low- (1.48 mM) and high- (1.20 mM) dose groups, after 12 weeks (p = 0.015). Significant differences between the high dose and placebo groups were also seen after both 6 and 12 weeks (p = 0.013 res. 0.014) as presented in Fig. 1B. After 6 weeks a significant change of calcium levels in the high dose probiotic group could be seen. The calcium concentration changed from 2.40 to 2.33 mM (p = 0,002) but after another 6 weeks the calcium concentration was normalized (see Table 2). One of the participants in the high dose groups showed increased CRP levels and low albumin throughout the intervention period, possibly indicating a potential systemic low-grade inflammation. Moreover, the albumin level, in the high dose group, decreased after 12 weeks from 44.44 to 42.80 g/L (p = 0.033), even when excluding the participant mentioned earlier. Furthermore, a trend towards decreased levels of the lipid parameters total cholesterol and LDL was observed in the high dose group; however, the decrease was not statistically significant. A similar tendency was also seen for cortisol.

**Table 2:** Chemical and biochemical characteristics of the placebo and probiotic groups low (LD) and high dose (HD).

Analyte	Placebo			Low dose			High dose		
	Baseline	6 weeks	12 weeks	Baseline	6 weeks	12 weeks	Baseline	6 weeks	12 weeks
CRP (mg/L)	1.49 ± 1.26	1.54 ± 1.34	2.11 ± 2.38	1.08 ± 0.77	1.34 ± 1.15	1.09 ± 0.62	3.22 ± 3.20	2.74 ± 2.61	2.59 ± 2.43
Creatinine (μM)	84.44 ± 9.06	84.56 ± 11.42	85.59 ± 9.64	86.07 ± 19.27	88.64 ± 11.84	86.50 ± 12.06	83.94 ± 11.45	85.60 ± 10.78	83.93 ± 13.04
Bilirubin (μM)	9.28 ± 2.82	9.44 ± 3.42	9.88 ± 3.46	13.0 ± 5.4	12.36 ± 4.8	13.71 ± 5.58	12.75 ± 9.07	10.40 ± 5.57	10.73 ± 5.28
Cystatin C DT-egfr	80.17 ± 7.08	80.99 ± 7.03	78.59 ± 6.76	79.0 ± 6.65	76.40 ± 6.08	75.99 ± 7.5	79.00 ± 6.65	76.40 ± 6.68	78.93 ± 7.50
Albumin (g/L)	43.28 ± 2.44	44.31 ± 2.87	43.47 ± 2.72	44.43 ± 2.56	45.36 ± 2.65	45.07 ± 3.36	44.44 ± 3.14	43.67 ± 3.58	42.80 ± 3.67
PSA (μg/L)	1.64 ± 1.21	1.41 ± 0.80	1.53 ± 0.83	2.03 ± 1.68	2.02 ± 1.71	1.88 ± 1.67	1.52 ± 1.13	1.35 ± 0.82	1.48 ± 1.50
SHBG (nmol/L)	46.78 ± 16.40	47.63 ± 18.54	44.38 ± 16.74	57.64 ± 13.72	47.5 ± 16.22	49.21 ± 16.85	40.56 ± 15.89	38.80 ± 14.52	39.25 ± 15.36
LH (IU/L)	5.17 ± 2.34	5.12 ± 2.30	4.94 ± 2.16	5.14 ± 3.32	4.01 ± 1.34	4.58 ± 1.69	4.74 ± 2.00	4.77 ± 1.97	4.83 ± 1.71
Testosterone (nM)	17.88 ± 6.16	17.84 ± 6.55	17.66 ± 8.34	18.74 ± 6.16	19.08 ± 5.5	18.9 ± 4.96	16.29 ± 6.84	15.53 ± 5.16	15.97 ± 5.91
DHEAS (μM)	4.81 ± 2.76	4.81 ± 2.82	4.95 ± 2.84	4.63 ± 2.48	4.5 ± 2.54	4.62 ± 2.53	5.18 ± 1.65	5.11 ± 1.81	4.91 ± 1.64
FSH (IU/L)	6.74 ± 3.30	6.95 ± 3.77	6.78 ± 3.84	4.56 ± 1.61	4.41 ± 1.64	4.61 ± 1.57	5.02 ± 2.62	5.19 ± 2.47	5.02 ± 2.28
TSH (mIU/L)	2.26 ± 1.42	2.62 ± 1.43	2.49 ± 1.39	2.54 ± 1.88	2.39 ± 1.16	2.48 ± 1.68	2.64 ± 1.32	2.66 ± 1.45	2.76 ± 1.56
T4 (nM)	15.94 ± 2.01	15.88 ± 2.25	15.29 ± 1.65	15.36 ± 1.82	15.57 ± 1.5	15.43 ± 1.79	15.75 ± 1.88	15.40 ± 1.80	15.20 ± 1.86
Cortisol (nM)	307.8 ± 75.2	332.1 ± 103.0	317.4 ± 96.0	347.2 ± 92.4	352.8 ± 103.8	346.1 ± 84.2	315.5 ± 93.1	307.1 ± 89.5	290.1 ± 103.3
Insulin (mIU/L)	15.82 ± 19.03	10.50 ± 7.85	9.82 ± 6.84	10.21 ± 6.46	11.71 ± 10.34	11.93 ± 7.29	12.19 ± 7.71	13.27 ± 7.26	11.87 ± 6.27
HbA1C (mmol/mol)	34.78 ± 3.28	34.94 ± 3.36	35.33 ± 3.15	34.21 ± 3.21	34.42 ± 3.02	35.6 ± 2.67	35.31 ± 2.73	35.80 ± 2.24	36.50 ± 2.53
Cholesterol (mM)	5.36 ± 1.33	5.46 ± 1.45	5.22 ± 1.36	5.47 ± 0.41	5.54 ± 0.41	5.54 ± 0.54	5.07 ± 0.95	4.80 ± 0.90	4.77 ± 0.94
LDL (mM)	3.52 ± 1.13	3.59 ± 1.42	3.39 ± 1.25	3.57 ± 0.46	3.78 ± 0.46	3.72 ± 0.41	3.25 ± 0.58	3.13 ± 1.00	3.10 ± 0.92
HDL (mM)	1.51 ± 0.32	1.52 ± 0.35	1.59 ± 0.32	1.59 ± 0.41	1.54 ± 0.30	1.54 ± 0.43	1.48 ± 0.51	1.47 ± 0.50	1.45 ± 0.50
Triglycerides (mM)	1.36 ± 0.73	1.52 ± 0.75	1.44 ± 0.65	1.40 ± 0.59	1.17 ± 0.43	1.48 ± 0.75	1.54 ± 0.44	1.22 ± 0.45	1.20 ± 0.41
Natrium (mM)	140.3 ± 1.8	140.1 ± 1.7	139.3 ± 1.9	140.9 ± 2.4	140.2 ± 2.7	139.8 ± 1.9	139.9 ± 2.0	140.3 ± 1.5	140.1 ± 2.3
Kalium (mM)	4.11 ± 0.33	4.08 ± 0.31	4.06 ± 0.2	4.09 ± 0.21	4.07 ± 0.19	4.11 ± 0.38	4.04 ± 0.37	4.03 ± 0.32	4.11 ± 0.25
Calcium (mM)	2.38 ± 0.06	2.36 ± 0.05	2.33 ± 0.06	2.40 ± 0.12	2.37 ± 0.08	2.40 ± 0.10	2.40 ± 0.09	2.33 ± 0.08	2.35 ± 0.10

Data concerning blood characteristics are presented in Table 3. Significant decreases of HB (p = 0.022), EVF (p = 0.027) and RBC (p = 0.023) levels, in the high dose group, were detected after 6 weeks. After 12 weeks the levels had however returned to baseline values. No further changes were observed.

**Table 3:** Blood work characteristics of the placebo, low- and high dose probiotic groups.

	Placebo			Low dose			High dose		
	Baseline	6 weeks	12 weeks	Baseline	6 weeks	12 weeks	Baseline	6 weeks	12 weeks
Erythrocytes (10^12^/L)	4.86 ± 0.38	4.83 ± 0.44	4.86 ± 0.35	4.99 ± 0.28	5.02 ± 0.30	5.06 ± 0.37	4.95 ± 0.47	4.89 ± 0.36	4.92 ± 0.40
EVF	0.48 ± 0.03	0.45 ± 0.03	0.45 ± 0.02	0.46 ± 0.02	0.46 ± 0.02	0.46 ± 0.03	0.45 ± 0.03	0.44 ± 0.02	0.45 ± 0.03
MCV (fL)	93.28 ± 2.78	93.50 ± 3.27	93.22 ± 3.08	92.21 ± 2.94	91.79 ± 3.08	91.71 ± 2.64	91.38 ± 4.19	91.07 ± 4.82	91.20 ± 4.36
MCHC (g/L)	31.11 ± 1.68	30.94 ± 1.48	30.88 ± 1.41	31.14 ± 1.39	30.71 ± 1.14	30.57 ± 1.02	30.50 ± 1.86	30.33 ± 1.68	30.33 ± 1.68
Hemoglobin (g/L)	150.5 ± 7.0	147.8 ± 9.3	149.6 ± 6.9	154.8 ± 5.7	153.1 ± 7.5	155.2 ± 8.6	151.7 ± 8.9	148.2 ± 6.5	149.5 ± 9.0
Leukocytes (10^9^/L)	5.59 ± 1.25	5.40 ± 1.28	5.22 ± 1.23	5.79 ± 1.09	5.81 ± 0.99	5.6 ± 1.15	6.17 ± 1.28	5.72 ± 0.98	5.89 ± 1.02
Lymphocytes (10^9^/L)	1.08 ± 0.12	1.83 ± 0.49	1.78 ± 0.59	2.27 ± 0.35	2.19 ± 0.62	2.21 ± 0.39	2.04 ± 0.46	1.94 ± 0.4	2.05 ± 0.52
Neutrophiles (10^9^/L)	2.82 ± 0.87	2.83 ± 0.40	2.70 ± 0.77	2.76 ± 0.80	2.69 ± 0.74	2.56 ± 0.65	2.32 ± 1.13	3.0 ± 0.83	3.16 ± 0.48
Thrombocytes (10^9^/L)	213.5 ± 51.2	217.4 ± 57.7	206.4 ± 52.4	248.5 ± 51.4	248.9 ± 43.7	258.0 ± 52.8	257.8 ± 47.5	255.4 ± 47.2	259.5 ± 47.1

The results from the AMS questionnaire (see Table 4) show that both the placebo and the high dose groups experience significant improvement (indicated by lower numbers) concerning somatic scores, after 6 and 12 weeks. Scores concerning both psychological and sexual matters in the low dose group indicate an improved state after 6 and 12 weeks. The total score for all groups were below 36 indicating no or mild symptoms only, consistent with a low testosterone level. In our case all study participants are well within the reference intervals given for the age group for the investigated parameters e.g., testosterone, SHGB, LH and FSH and thereby not considered deficient in these parameters [20]. The total mean score in both the placebo and the low dose group decreased by 15 % and 13 % respectively compared to the high dose group that only decreased by 4 %.

**Table 4:** AMS questionnaire characteristics of the placebo, low- and high dose probiotic groups.

	Placebo			Low dose			High dose		
	Baseline	6 weeks	12 weeks	Baseline	6 weeks	12 weeks	Baseline	6 weeks	12 weeks
Somatic	12.89 ± 3.98	10.82 ± 3.30	10.12 ± 2.93	10.26 ± 2.6	9.57 ± 2.34	10.14 ± 2.77	13.75 ± 4.60	11.88 ± 3.59	12.20 ± 3.91
Psychologic	7.06 ± 2.62	6.24 ± 2.36	5.94 ± 1.75	6.57 ± 1.50	5.95 ± 1.49	5.71 ± 0.99	6.81 ± 2.17	6.94 ± 2.72	6.87 ± 2.29
Sexual	10.78 ± 3.81	10.29 ± 4.47	9.24 ± 3.61	11.43 ± 3.63	9.36 ± 3.18	9.43 ± 3.92	10.69 ± 3.26	10.06 ± 2.89	10.67 ± 3.37
Total	30.72 ± 8.84	27.35 ± 9.04	25.29 ± 7.60	28.54 ± 7.37	24.40 ± 6.20	24.73 ± 7.15	31.25 ± 8.34	28.88 ± 7.39	29.73 ± 7.18

During the study, no self-reported adverse events were observed besides some general comments from a few individuals in all groups, concerning increased flatulence.

## 4 Discussion

Age-related testosterone deficiency is a factor of concern regarding bone loss in elderly men, therefore maintaining adequate testosterone levels is critical for bone health. It should be mentioned that all patients got a daily dose of vitamin D (400 IU). Effects of vitamin D supplementation has been shown to increase testosterone in a group of healthy overweight men who underwent a weight reduction program and received 3332 IU vitamin D3 daily for one year [21]. Most likely the increase found in the study could be explained by the fact that reduction in fat will release testosterone into the circulation and thereby increase the serum concentration. Two other studies show no increase in testosterone levels upon supplementation with vitamin D3 ranging from 4200 IU to 40,000 IU over a time of 4–12 month [22,23].

In the present study we set out to investigate the effect of probiotic supplementation of *L. reuteri* on testosterone levels in ageing men. It has recently been shown that supplementation of the LR strain could significantly reduce bone loss in older women with low bone mineral density [13]. Preclinical evidence show that the observed effect of *L. reuteri* on bone density is to a large extent driven by effects on T cells [[24], [25], [26]] but it is reasonable to expect that the effects in postmenopausal women could be hormone related, and therefore LR could potentially influence testosterone in older “post andropausal” men where testosterone is in decline [2]. *L. reuteri* was shown to have multiple effects on different hormones in several different mouse studies. One study observed that when mice were administered this probiotic daily for 30 days there was an increase in serum testosterone levels, increase in Langerhans cells and testes size compared to those administered a placebo product [14]. Two studies reveal the effects of probiotic supplementation on hormones in women, the first relates to individuals diagnosed with polycystic ovary syndrome, accompanied with increased levels of SHBG [27]. The second study regarded perimenopausal, and postmenopausal women supplemented with a multi-species probiotic product containing 8 species of *Bifidobacterium* sp and *Lactobacillu*s; a total dose of 2,5 × 10^9^ CFU three times per day for five weeks resulted in a significant increase of FSH [28].

The present study did not show any effects on testosterone levels in the investigated population, regardless of the LR supplementation dose. The present study has included only healthy individuals, with no known deficiencies in testosterone and have not seen any effect on the levels of this hormone. It cannot be excluded that supplementation with probiotics, could have an effect in individuals with low testosterone production. No significant changes could be detected using the AMS-score analysis, although some improvements were reported both on somatic-, physiological- and sexual scores. In the high dose group, no total changes in the score were seen. In the placebo group, there is a tendency of reduced score over time in all parameters measured. For the low dose group, there is a tendency for psychological- and sexual scores to decrease over time. This could perhaps be due to known “placebo effects” leading to a feeling of increased quality of life, due to the participation in a study. The reported improvement in the low dose group is not correlating to any other investigated parameter. However, since the “placebo effects”, manifested as a lowering of scores could not be seen in the high-dose group, this needs further investigation. The results could perhaps mirror the presence of slightly increased CRP values, compared to the low dose and placebo groups, indicating a low-grade inflammation (see Table 2). It could also be hypothesized that supplementation for a longer time period would be necessary [27]. Furthermore, it might be reasonable to assume that an effect on hormones might be found in those participants with a hormone deficient condition. Interestingly, a study published in 2021 saw a significant increase in follicle stimulating hormone (FSH) in perimenopausal women after 5 weeks supplementation with *L. plantarum* 229v [28].

Several previous studies have shown that, in both animal models [[29], [30], [31], [32]] as well as in human studies [33,34], the lipid profile is affected by supplementation of various probiotic bacteria, including *L. reuteri* [35,36]. In the case of L. reuteri intervention, both in man and mice, a reduction of cholesterol could be seen, however not in triglyceride concentrations. In the case of this study, we could not detect any significant change in cholesterol levels, although there was a tendency of decrease in the high dose group, concerning both total cholesterol, LDL and HDL. One may speculate that this is a result from a limited number of participants, completing the study and the intervention length. The effect on triglyceride-lowering capability and apparent lack of effects on cholesterol may be due to differences in effect on metabolism. It has been suggested that the cholesterol-lowering effects of L. *reuteri* NCIMB 30242 may be couple to its capability of decoupling bile acids without having any impact on triglyceride levels. Reduction of only triglycerides and not on cholesterol may suggest a strain specific effect on lipid metabolism more in line with findings in other L. *reuteri* strains [37]. Interestingly, Dixon et al. presented a systematic review on the efficacy of probiotics showing that statistically significant changes were seen in improvements of blood-pressure, cholesterol, LDL and HDL, however no significant change was observed in the outcome of triglycerides [38].

The only significant change regarding the investigated parameters, found in this study, is a triglyceride concentration decrease in the high dose group, both after 6 and 12 weeks of supplementation. A paper by Ahn et al. is, to our knowledge the only previous study, describing lowering of triglycerides upon supplementation with dual probiotic strains in humans [39]. The lowering effect on triglycerides is especially interesting, since the availability of suitable drugs, targeting triglycerides is limited. The changes in triglycerides are within the normal range but shows promising future treatment options that are appealing since probiotics are not known to have side-effects likely affecting the intended groups in need of such intervention.

Finally, it should also be noted that both the recruitment and the study itself was performed during the time of the COVID-19 pandemic. The pandemic probably caused significant constraints on the lifestyle of the participants, with restrictions on social interactions as an increased preference for out-door activities. This may have had an impact on the study results. The effects of the pandemic on the recording of mental scores are well documented and are both due to direct effects of the infection as well as the social restrictions enforced during the pandemic [40,41]. The study was powered to meet the expected shift of the primary outcome, testosterone concentrations, and no other investigated analytes. Therefore, the number of subjects could be too low to meet the requirement for statistical significance. It should also be mentioned that effects on testosterone as response to *L*. *reuteri* administration has not previously been investigated in man and the study is therefore a pilot study and the number of subjects can therefore be too low also in the primary variable.

## 5 Conclusion

In conclusion, the present study does not support the hypothesis that a probiotic supplementation with *L. reuteri* ATCC PTA 6475 can increase testosterone levels in ageing men. The AMS questionnaire indicates that a low dose improves the notion of psychological and sexual well-being. However, the supplementation does have a significant effect, decreasing triglyceride levels and this indicative data warrants further investigations into this area.

## CRediT authorship contribution statement

**Lennart Ljunggren:** Conceptualization, Formal analysis, Writing – original draft, Writing – review & editing. **Eile Butler:** Conceptualization, Investigation, Project administration. **Jakob Axelsson:** Project administration, Validation, Writing – review & editing. **Mikael Åström:** Formal analysis, Methodology. **Lars Ohlsson:** Conceptualization, Formal analysis, Project administration, Writing – original draft, Writing – review & editing.

## Declaration of competing interest

The authors declare the following financial interests/personal relationships which may be considered as potential competing interests:

Jakob Axelsson is an employee of Biogaia AB. The other authors declare that they have no known financial interest or personal relationships that could have appeared to influence the work reported in this paper.

The manuscript has been written and produced without any use of AI.

## References

[1] Handelsman D.J., Sikaris K., Ly L.P. (2016). Estimating age-specific trends in circulating testosterone and sex hormone-binding globulin in males and females across the lifespan. Ann. Clin. Biochem..

[2] Feldman H.A. (2002). Age trends in the level of serum testosterone and other hormones in middle-aged men: longitudinal results from the Massachusetts male aging study. J. Clin. Endocrinol. Metab..

[3] Travison T.G. (2007). The relative contributions of aging, health, and lifestyle factors to serum testosterone decline in men. J. Clin. Endocrinol. Metab..

[4] Trost L.W., Mulhall J.P. (2016). Challenges in testosterone measurement, data interpretation, and methodological appraisal of interventional trials. J. Sex. Med..

[5] Bassil N., Alkaade S., Morley J.E. (2009). The benefits and risks of testosterone replacement therapy: a review. Therapeut. Clin. Risk Manag..

[6] Bhasin S. (2001). Testosterone dose-response relationships in healthy young men. Am. J. Physiol. Endocrinol. Metab..

[7] Bravo J.A. (2011). Ingestion of Lactobacillus strain regulates emotional behavior and central GABA receptor expression in a mouse via the vagus nerve. Proc. Natl. Acad. Sci. U. S. A..

[8] Kelly J.R. (2017). Lost in translation? The potential psychobiotic Lactobacillus rhamnosus (JB-1) fails to modulate stress or cognitive performance in healthy male subjects. Brain Behav. Immun..

[9] Cleusix V. (2007). Inhibitory activity spectrum of reuterin produced by Lactobacillus reuteri against intestinal bacteria. BMC Microbiol..

[10] Savino F. (2010). Lactobacillus reuteri DSM 17938 in infantile colic: a randomized, double-blind, placebo-controlled trial. Pediatrics.

[11] Urbanska M., Gieruszczak-Bialek D., Szajewska H. (2016). Systematic review with meta-analysis: Lactobacillus reuteri DSM 17938 for diarrhoeal diseases in children. Aliment. Pharmacol. Ther..

[12] Poutahidis T. (2013). Microbial symbionts accelerate wound healing via the neuropeptide hormone oxytocin. PLoS One.

[13] Nilsson A.G. (2018). Lactobacillus reuteri reduces bone loss in older women with low bone mineral density: a randomized, placebo-controlled, double-blind, clinical trial. J. Intern. Med..

[14] Poutahidis T. (2014). Probiotic microbes sustain youthful serum testosterone levels and testicular size in aging mice. PLoS One.

[15] Matsumoto A.M. (2002). Andropause: clinical implications of the decline in serum testosterone levels with aging in men. J Gerontol A Biol Sci Med Sci.

[16] Stanworth R.D., Jones T.H. (2008). Testosterone for the aging male; current evidence and recommended practice. Clin. Interv. Aging.

[17] Spinler J.K. (2008). Human-derived probiotic Lactobacillus reuteri demonstrate antimicrobial activities targeting diverse enteric bacterial pathogens. Anaerobe.

[18] Emmelot-Vonk M.H. (2011). Low testosterone concentrations and the symptoms of testosterone deficiency according to the Androgen Deficiency in Ageing Males (ADAM) and Ageing Males' Symptoms rating scale (AMS) questionnaires. Clin. Endocrinol..

[19] Heinemann L.A. (2003). The Aging Males' Symptoms (AMS) scale: update and compilation of international versions. Health Qual. Life Outcome.

[20] Bjerner J. (2009). Reference intervals for serum testosterone, SHBG, LH and FSH in males from the NORIP project. Scand. J. Clin. Lab. Invest..

[21] Pilz S. (2011). Effect of vitamin D supplementation on testosterone levels in men. Horm. Metab. Res..

[22] Heijboer A.C. (2015). Vitamin D supplementation and testosterone concentrations in male human subjects. Clin. Endocrinol..

[23] Jorde R. (2013). Supplementation with vitamin D does not increase serum testosterone levels in healthy males. Horm. Metab. Res..

[24] Britton R.A. (2014). Probiotic L. reuteri treatment prevents bone loss in a menopausal ovariectomized mouse model. J. Cell. Physiol..

[25] Collins F.L. (2019). Beneficial effects of Lactobacillus reuteri 6475 on bone density in male mice is dependent on lymphocytes. Sci. Rep..

[26] McCabe L.R. (2013). Probiotic use decreases intestinal inflammation and increases bone density in healthy male but not female mice. J. Cell. Physiol..

[27] Arab A. (2022). Effects of probiotic supplementation on hormonal and clinical outcomes of women diagnosed with polycystic ovary syndrome: a double-blind, randomized, placebo-controlled clinical trial. J. Funct.Foods.

[28] Szydłowska I. (2021). Effects of probiotics supplementation on the hormone and body mass index in perimenopausal and postmenopausal women using the standardized diet. A 5-week double-blind, placebo-controlled, and randomized clinical study. Eur. Rev. Med. Pharmacol. Sci..

[29] Aderiye B., Laleye S., Odeyemi A. (2007). Hypolipidemic effect of Lactobacillus and Streptococcus species from some Nigerian fermented foods. Res. J. Microbiol..

[30] Al-Ajeeli F.S., Flayyih M.T., Alkhazrajy L.A. (2013). Anti obesity and lipid-lowering effect of Lactobacillus spp. as probiotic on the obese rat. Iraqi J. Sci..

[31] Fathi M. (2013). Effects of Lactobacillus cultures as probiotic on blood parameters, plasma enzymes activities and mortality in broiler chicken. Res. J. Anim. Sci..

[32] Mansoub N.H. (2010). Effect of probiotic bacteria utilization on serum cholesterol and triglycerides contents and performance of broiler chickens. Global Vet..

[33] Cicero A.F.G. (2021). Impact of a short-term synbiotic supplementation on metabolic syndrome and systemic inflammation in elderly patients: a randomized placebo-controlled clinical trial. Eur. J. Nutr..

[34] Wu Y. (2017). Effect of probiotic Lactobacillus on lipid profile: a systematic review and meta-analysis of randomized, controlled trials. PLoS One.

[35] Jones M.L., Martoni C.J., Prakash S. (2012). Cholesterol lowering and inhibition of sterol absorption by Lactobacillus reuteri NCIMB 30242: a randomized controlled trial. Eur. J. Clin. Nutr..

[36] Lu M. (2022). Prevention of high-fat diet-induced hypercholesterolemia by Lactobacillus reuteri Fn041 through promoting cholesterol and bile salt excretion and intestinal mucosal barrier functions. Front. Nutr..

[37] Werlinger P. (2022). Lactobacillus reuteri MJM60668 prevent progression of non-alcoholic fatty liver disease through anti-adipogenesis and anti-inflammatory pathway. Microorganisms.

[38] Dixon A. (2020). Efficacy of probiotics in patients of cardiovascular disease risk: a systematic review and meta-analysis. Curr. Hypertens. Rep..

[39] Ahn H. (2015). The triglyceride-lowering effect of supplementation with dual probiotic strains, Lactobacillus curvatus HY7601 and Lactobacillus plantarum KY1032: reduction of fasting plasma lysophosphatidylcholines in nondiabetic and hypertriglyceridemic subjects. Nutr. Metabol. Cardiovasc. Dis..

[40] Penninx B.W. (2022). How COVID-19 shaped mental health: from infection to pandemic effects. Nat. Med..

[41] Webb L.M., Chen C.Y. (2022). The COVID‐19 pandemic's impact on older adults' mental health: contributing factors, coping strategies, and opportunities for improvement. Int. J. Geriatr. Psychiatr..

